# Systematic Review of Pharmacogenetic Factors That Influence High-Dose Methotrexate Pharmacokinetics in Pediatric Malignancies

**DOI:** 10.3390/cancers13112837

**Published:** 2021-06-07

**Authors:** Zachary L. Taylor, Jesper Vang, Elixabet Lopez-Lopez, Natanja Oosterom, Torben Mikkelsen, Laura B. Ramsey

**Affiliations:** 1 Department of Pharmacology and Systems Physiology, University of Cincinnati, Cincinnati, OH 45267, USA; taylorzl@mail.uc.edu; 2 Division of Research in Patient Services, Cincinnati Children’s Hospital Medical Center, Cincinnati, OH 45229, USA; 3 Division of Clinical Pharmacology, Cincinnati Children’s Hospital Medical Center, Cincinnati, OH 45229, USA; 4 Department of Health Technology, Technical University of Denmark, 2800 Lyngby, Denmark; jvan@dtu.dk; 5 Paediatric Oncology Research Laboratory, University Hospital of Copenhagen, Rigshospitalet Blegdamsvej 9, 2100 Copenhagen, Denmark; 6 Department of Genetics, Physical Anthropology and Animal Physiology, Faculty of Science and Technology, University of the Basque Country, UPV/EHU, 48940 Leioa, Spain; elixabet.lopez@ehu.eus; 7 Pediatric Oncology Group, BioCruces Bizkaia Health Research Institute, 48903 Barakaldo, Spain; 8 Princess Máxima Center for Pediatric Oncology, 3720 Utrecht, The Netherlands; n.oosterom@prinsesmaximacentrum.nl; 9 Department of Pediatric Oncology, Aarhus University Hospital, 8200 Aarhus, Denmark; torben.mikkelsen@clin.au.dk

**Keywords:** methotrexate, pharmacokinetics, pharmacogenetics, pediatrics, acute lymphoblastic leukemia, osteosarcoma, lymphoma

## Abstract

**Simple Summary:**

High-dose methotrexate is commonly used to treat several types of cancer. While effective, elimination of high-dose methotrexate from the body is highly variable and delayed elimination can lead to serious and sometime life-threatening adverse events. There have been many clinical studies to better understand how genetics influence the variability in methotrexate elimination, mostly through candidate gene studies and three genome-wide association studies. Unfortunately, there are conflicting results and some studies lack the appropriate replication and validation needed to confirm their effects on methotrexate elimination. Therefore, the purpose of this systematic review was to summarize all of the germline pharmacogenetic association studies of genetic associations influencing high-dose methotrexate elimination in children with cancer.

**Abstract:**

Methotrexate (MTX) is a mainstay therapeutic agent administered at high doses for the treatment of pediatric and adult malignancies, such as acute lymphoblastic leukemia, osteosarcoma, and lymphoma. Despite the vast evidence for clinical efficacy, high-dose MTX displays significant inter-individual pharmacokinetic variability. Delayed MTX clearance can lead to prolonged, elevated exposure, causing increased risks for nephrotoxicity, mucositis, seizures, and neutropenia. Numerous pharmacogenetic studies have investigated the effects of several genes and polymorphisms on MTX clearance in an attempt to better understand the pharmacokinetic variability and improve patient outcomes. To date, several genes and polymorphisms that affect MTX clearance have been identified. However, evidence for select genes have conflicting results or lack the necessary replication and validation needed to confirm their effects on MTX clearance. Therefore, we performed a systematic review to identify and then summarize the pharmacogenetic factors that influence high-dose MTX pharmacokinetics in pediatric malignancies. Using the PRISMA guidelines, we analyzed 58 articles and 24 different genes that were associated with transporter pharmacology or the folate transport pathway. We conclude that there is only one gene that reliably demonstrates an effect on MTX pharmacokinetics: *SLCO1B1*.

## 1. Introduction

Methotrexate (MTX) is a folate analog that has long been administered at high doses to effectively treat pediatric and adult malignancies such as acute lymphoblastic leukemia (ALL), lymphoma, and osteosarcoma [1,2,3,4,5]. MTX, in its polyglutamated form, is an effective chemotherapeutic agent that competitively inhibits the dihydrofolate reductase, a key enzyme responsible for the reduction reaction of dihydrofolate to tetrahydrofolate [6]. Such inhibition drains the cells of necessary tetrahydrofolate, thus disrupting the de novo biosynthesis of purines and pyrimidines and preventing the formation of necessary one-carbon donors needed for DNA methylation [7]. High-dose MTX has contributed to the dramatically increased survival rates for pediatric patients with ALL [8,9,10]. Despite this improvement, there remains a troubling occurrence of high-dose MTX-induced toxicities [11,12] that often results from variability in MTX pharmacokinetics (PK). Numerous clinical studies have investigated this variability in MTX PK in relation to treatment response or toxicity. Since protocols with high-dose MTX treatment include frequent monitoring, extensive amounts of clinical data are often collected during each patients’ treatment. Tests of renal function are of particular importance, given that MTX is primarily renally eliminated [11]. A decrease or delay in MTX elimination as a result of damage to the renal tubules can cause prolonged systemic exposure to MTX. Renal dysfunction can cause a subsequent reduction in both renal absorption and elimination of MTX [11,12]. Conversely, the liver plays a key role in the metabolic conversion of MTX into 7-hydroxyMTX by aldehyde oxidase. However, in high-dose MTX infusions, reports indicate that 55–80% of MTX is eliminated as unchanged drug in the urine [13]. Hepatic elimination of MTX is only estimated to account for 10% of MTX elimination [14].

PK findings from clinical studies can lead to improvements in therapeutic outcomes through amendments to clinical protocols [5,15] or the development of clinical decision support tools [16]. It was recently shown that >90% of persons have an actionable drug-gene polymorphism [17,18]. A plethora of clinical studies have been including pharmacogenetics in an effort to describe more of the variability in MTX PK and improve therapeutic outcomes. To date, pharmacogenetic studies have identified several genes that contribute to the vast variability in MTX PK and primarily fall under folate pathway genes (*MTHFR, MTR, MTRR,* and *DHFR*) or PK/transporter genes (*SLCO1B1, SLC19A1, OAT1,* and *OAT3*). However, trying to use these data in a clinically meaningful way can be challenging due to the sheer volume of pharmacogenetic data that exist. Additionally, there is inconsistent and contrasting evidence available for some genes and genetic variants, which can complicate their clinical utility, while others lack the appropriate replication and validation needed to be reliable predictors of MTX PK. A great resource that catalogs the pharmacogenetic literature is the Pharmacogenomics Knowledge Base (www.pharmgkb.org, accessed on 27 May 2021), but for MTX, it does not readily distinguish between high-dose and low-dose indications, where we know the dose impacts the PK and pharmacodynamics, and therefore may have different pharmacogenetic associations.

Therefore, the objective of this systematic review was to identify and then provide a comprehensive overview of the available pharmacogenetic factors that influence high-dose MTX PK in pediatric malignancies. Herein, we discuss the use of the PRISMA guidelines to select and summarize genome-wide association studies and candidate gene studies that evaluated the association between genetic polymorphisms and MTX PK in pediatric patients with acute lymphoblastic leukemia, lymphoma, or osteosarcoma.

## 2. Materials and Methods

### 2.1. Eligibility Criteria

We used the PRISMA guidelines for systematic review [19]. Studies were included if they:

- Evaluated the association between genetic polymorphisms and MTX PK;
- Included pediatric patients
- With ALL, lymphoma, or osteosarcoma.

Studies were excluded if they:

- Only investigated the PK of MTX (no genetics),
- Only investigated the pharmacogenetics of MTX on toxicity (no PK),
- Only investigated patients with other malignancies,
- Did not include pediatric patients (only include adult populations), and
- Were not published in English.

### 2.2. Search Strategy

We searched PubMed and Scopus on November of 2020 and February of 2021. We used a combination of keywords and medical subject headings that focused on five key search criteria: (1) MTX; (2) PK; (3) pharmacogenetics; (4) pediatrics; (5) ALL, lymphoma, and osteosarcoma. The full list of search queries for PubMed and Scopus can be found in the Supplementary Materials, Table S1.

### 2.3. Data Collection

Z.L.T and L.B.R independently assessed the eligibility of the studies by reviewing abstracts. Full texts were considered on an “as-needed” basis. Conflicts between Z.L.T and L.B.R were resolved by discussion. Z.L.T extracted data from the included studies using the available full text: study type (GWAS or candidate gene study), patient population (number of patients, indication), treatment methods (treatment protocol, dosages, course quantity), study design (quantification method for MTX, method for genotyping), PK endpoints (clearance, concentrations at certain times after a dose, delayed MTX elimination), and genes studied.

### 2.4. Qualitative Data Analysis

For each article, the selected gene(s) and associated polymorphism(s) was/were noted along with the primary PK endpoint, and the directionality of the effect. After all included articles were annotated, Z.L.T determined whether the overall effect of each gene and associated polymorphisms decreased MTX clearance, increased MTX exposure, and/or increased MTX concentrations along with a short synopsis of the available, published data. Articles that presented conflicting evidence were noted and taken into consideration when determining the overall effect of the gene and polymorphism. Genes were then assigned to a gene table (transporter or folate pathway), and commonly studied polymorphisms were presented. The full list of genes and polymorphisms can be found in the Supplementary Materials, Table S2.

## 3. Results

### 3.1. Study Selection

**Figure 1:**
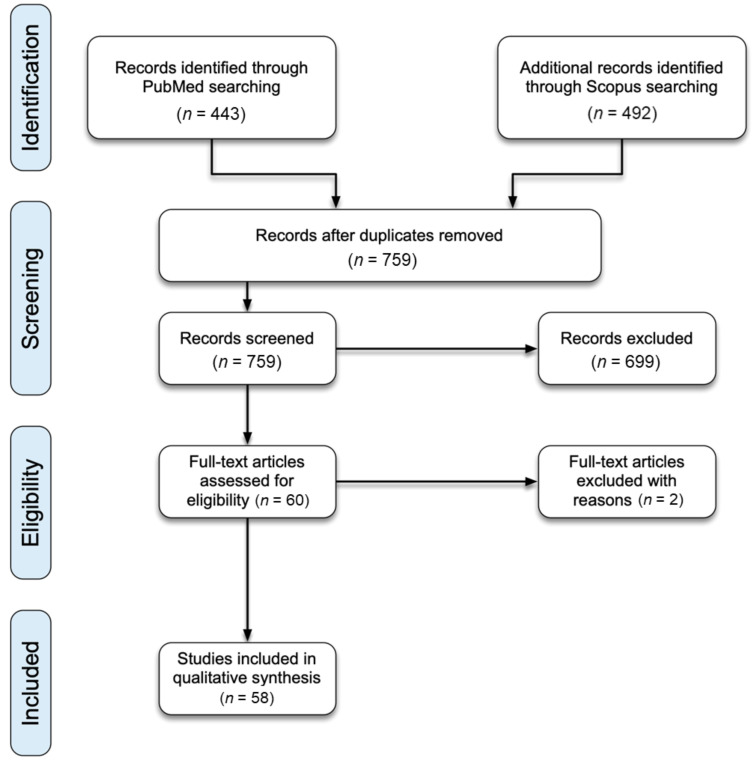
PRISMA diagram for systematic literature review.

Figure 1 shows the PRISMA flow diagram for this literature review. The final PubMed search performed in February 2021 found 443 articles. The final Scopus search performed in February 2021 found 492 articles. After duplications were removed, 759 articles remained. The initial abstract screening process excluded 699 articles. Full-text of the remaining 60 articles were assessed. Upon full-text review, two additional articles were excluded due to a lack of pharmacogenetic associations with MTX PK, resulting in a final total of 58 articles for qualitative analysis.

### 3.2. Study Characteristics

The 58 studies analyzed pharmacogenetic associations on MTX PKs from 9695 patients. Three studies were GWAS and 55 were candidate gene studies. ALL was the most commonly studied malignancy, with 51 of the 58 articles reporting on pediatric patients with ALL, followed by five studies reporting on pediatric patients with osteosarcoma, and three studies reporting on pediatric patients with lymphoma. One study reported on both patients with ALL and patients with lymphoma.

Of the reported studies, Sequenom MassARRAY was most commonly used for genotyping and fluorescence polarization immunoassay was most commonly used to detect and quantify MTX concentrations.

### 3.3. Association between Genetic Polymorphisms and MTX PKs

#### 3.3.1. Transporter Genes

The following information is summarized and presented in Table 1.

##### ABCB1

**Figure 2:**
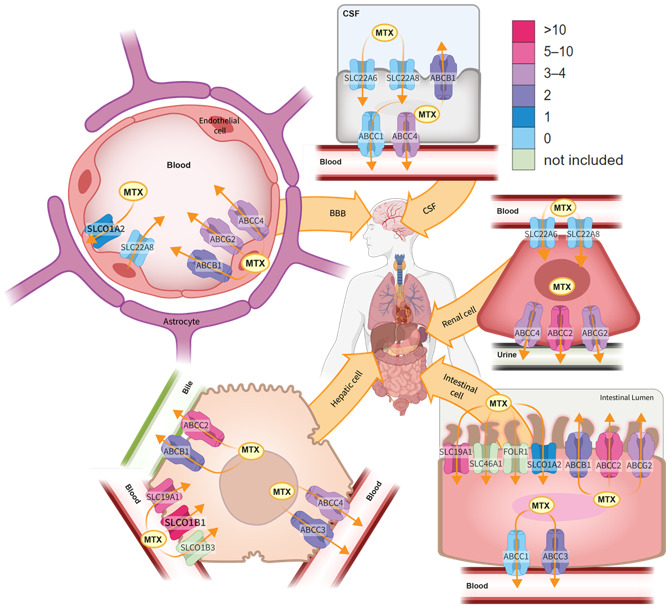
Transporter genes involved in methotrexate pharmacology. Transporters of similar function are grouped by shape. The color scheme for the transporters reflects the number of studies that found a significant pharmacogenetic association with MTX exposure. BBB: blood–brain barrier; CSF: cerebrospinal fluid; FOLR1: Folate Receptor Alpha; MTX: methotrexate.

ATP-binding cassette subfamily B member 1 (*ABCB1*) encodes an ATP-dependent drug efflux pump involved in multidrug resistance, which is expressed in liver, kidney and gastrointestinal tract [56] (Figure 2). The association of polymorphisms in this gene with MTX PK has been studied in 13 different studies. The most commonly studied SNP was rs1045642, which was included in eight studies; while four studies identified a decrease in MTX clearance associated with the rs1045642T allele [25,26,34,57], only one of them reached the statistical significance threshold [26], and the other four studies did not find an effect of the SNP [20,27,35,38]. On the other hand, the rs9282564T allele was significantly associated with MTX AUC and Cmax [20]; however, it was not associated with MTX PK in the other study in which it was analyzed [21].

##### ABCG2

ATP-binding cassette subfamily G member 2 (*ABCG2*) encodes another ATP-binding cassette (ABC) transporter involved in multidrug resistance and is primarily expressed in the gastrointestinal tract [56] (Figure 2). Polymorphisms in this gene have been included in 13 different studies in order to determine their role in MTX PK. The most frequently studied SNP was rs2231142, which was analyzed in six studies. The variant A allele was significantly associated with decreased MTX clearance in two studies [20,26], while the same directionality was observed in another study, without reaching significance [25], and no association was identified in the other three studies [23,24,27]. In addition, three SNPs (rs12505410, rs13120400, and rs13137622) were significantly associated with MTX PK in a single study [22]. Nevertheless, since this is the only article in which they were studied, additional studies would be needed to confirm those associations.

##### ABCC2

ATP-binding cassette subfamily C member 2 (*ABCC2*) encodes an ABC transporter involved in multidrug resistance that is highly expressed in liver and kidney, where it is responsible for the efflux of different drugs [56] (Figure 2). Polymorphisms in this gene have been analyzed in 16 different studies. Among them, three SNPs (rs717620, rs3740065, and rs3740066) have been significantly associated with MTX PK but with conflicting results. The most commonly studied was rs717620, which was included in 13 studies [20,22,23,27,28,29,30,31,32,33,34,35,36]. While three of the studies supported an association of the variant T allele with increased MTX clearance [28,29,32], the other two suggested an association with decreased MTX clearance [30,31], and the remaining studies did not show any effect. Similarly, rs3740065 was investigated in five studies [22,28,29,30,31], and, while three studies supported an association of the variant C allele with decreased MTX clearance [29,30,31], another study suggested that the variant C allele increased MTX clearance [28]. Finally, rs3740066 was analyzed in three studies [20,27,29], one of them supporting an association with increased clearance [20], another showing a non-significant association with decreased clearance [29], and the remaining reporting no effect.

##### ABCC3

ATP-binding cassette subfamily C member 3 (ABCC3) is another multidrug resistance protein that is ubiquitously expressed [56] (Figure 2). Polymorphisms in this gene have been included in seven different studies. Two SNPs were significantly associated with MTX PK, rs4793665 [20], which was not associated with MTX PK in the other study in which it was studied [36], and rs9895420, which was reported in a single study [36].

##### ABCC4

ATP-binding cassette subfamily C member 4 (*ABCC4*) is a multidrug resistance protein from the ABC superfamily. Eight studies have investigated polymorphisms in this gene in relation to MTX PK. There is moderate evidence to suggest that four SNPs (rs10219913, rs7317112, rs9516519, and rs868853) might alter MTX clearance. Two studies supported the association of the rs10219913 variant C allele and the rs7317112 variant G allele with decreased MTX clearance [28,29]. Conversely, two of the three studies that analyzed rs868853 supported an association of the C variant allele with increased MTX clearance [31,37]. Finally, regarding rs9516519, three studies have analyzed this SNP [28,29,31]. The variant G allele was significantly associated with increased clearance one of the studies [29], and a similar tendency was observed in another one [31].

##### ABCC1, ABCC5 and ABCC10

*ABCC1*, *ABCC5* and *ABCC10* are multi-specific organic anion pumps and part of the MRP subfamily that are ubiquitously expressed throughout the body [56] (Figure 2). Polymorphisms in the remaining ABC transporters *ABCC1*, *ABCC5*, and *ABCC10* have been studied in seven, four and four studies, respectively. However, none of the studies reported statistically significant associations with MTX PK.

##### SLC19A1

**Figure 3:**
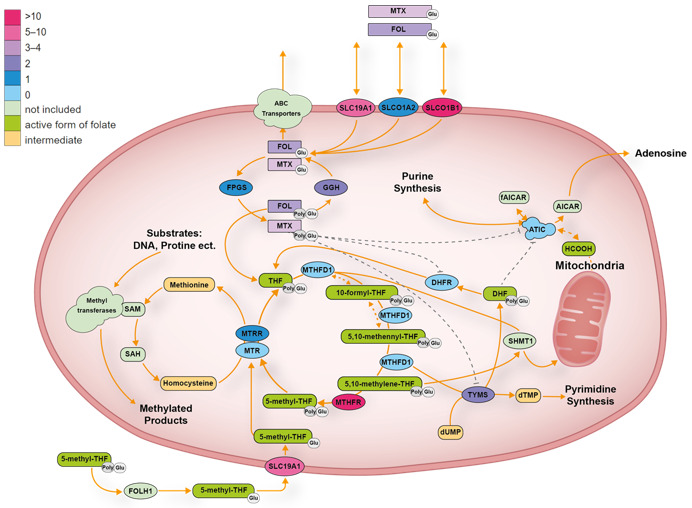
Folate transport pathway. The color scheme for the folate pathway genes reflects the number of studies that found a significant pharmacogenetic association with MTX exposure. AICAR: 5-aminoimidazole-4-carboxamide ribonucleotide; DHF: dihydrofolate; dUMP: deoxyuridine monophosphate; dTMP: deoxythymidine monophosphate; fAICAR: 5-formamidoimidazole-4-carboxamide ribonucleotide; FOL: folate; Glu: glutamated; FOLH1: N-acetyl-L-aspartyl-L-glutamate peptidase; I HCOOH: formic acid; MTX: methotrexate; Poly: prefix, meaning more than one, SAM: S-adenosyl methionine, SAH: S-adenosyl-l-homocysteine; SHMT1: serine hydroxymethyltransferase 1; THF: tetrahydrofolate.

The solute carrier family 19 member 1 or reduced folate carrier 1 is a ubiquitously and robustly expressed transporter in humans that mediates the uptake of endogenous reduced folates and anti-folate xenobiotics [56] (Figure 2 and Figure 3). *SLC19A1* also plays a key role in folate homeostasis. Due to its associations with both MTX transport and folate metabolism, *SLC19A1* was highly studied, appearing in 24 gene candidate studies with 20 different SNPs. Seven of the 24 gene candidate studies found a significant association between *SLC19A1* and MTX PK. The rs1051266 (G80A, formerly known as rs61510559) was the most abundantly studied and is a common non-synonymous polymorphism resulting in a substitution of a histidine for an arginine. Of the 21 studies that reported on rs1051266, five studies found a significant association [40,41,42,43,45] with MTX PK. Four of the five reported findings agree that the variant A allele is associated with decreased MTX clearance. This directionality was supported by two additional studies [26,29], but both failed to reach their set significance thresholds. Conversely, several more studies [20,21,22,23,25,27,28,33,34,35,38,39,45,46,47], including the aforementioned study [45], demonstrated that the variant A allele is associated with increased MTX clearance; all but one [45] reported finding of this directionality failed to reach their set significance thresholds. The rs1131596 variant was reported by two studies [29,38]. Both studies did not find a significant association between rs1131596 and MTX PK. Lastly, the rs3788200 variant was reported by two studies [29,38]. Both studies did not find a significant association between rs378820 and MTX PK. The three GWAS did not report a significant association between polymorphisms of *SLC19A1* and MTX PK.

##### SLC22A6 and SLCO22A8

The *SLC22A6* and *SLC22A8* solute carriers encode the organic anion transporters 1 and 3 (OAT1 and OAT3) that facilitate transport of many different organic anions including urate, probenecid and proton pump inhibitors [58,59,60]. In vitro studies have confirmed that the OAT1 and OAT3 transporters have affinity for MTX and that the cellular uptake of MTX can be inhibited by administration of other organic anions (e.g., probenecid and proton pump inhibitors) [58,59,60,61]. The OAT1 and OAT3 proteins are located on the basolateral membrane of the renal tubules cells, where they facilitate uptake of MTX from the blood into the cells [56] (Figure 2). Several non-synonymous SNPs with low allele frequency have been identified in the coding region of *SLC22A8* and shown to alter the in vitro uptake of MTX but the allele frequencies of these SNPs are low [62]. Animal studies with *Oat3* knock-out mice indicate that the Oat3 transporter is involved in the plasma clearance of MTX and reduced folates but also suggest that this is not the most important route of MTX elimination [63].

Polymorphisms in *SLCO22A6* and *SLCO22A8* were not associated with MTX PK in any of the genome-wide association studies [49,50,51], and only two candidate gene studies included SNPs from the two genes [21,29]. Lopez-Lopez et al. found an association between the 72-h plasma MTX > 0.2 µM and two *SLC22A6/SLC22A8* haplotypes but this was not statistically significant after adjusting for multiple comparisons [29]. In another candidate gene study, the variant C allele in rs4149183 in *SLC22A8* was associated with renal toxicity (serum creatinine level > 100 µmol/L) but not plasma concentrations of MTX or 7-OH-MTX [21]. Most of the 15 studied SNPs in the two candidate gene studies were located in introns or upstream to the genes.

##### SLCO1A2

The solute carrier organic anion transporter family member 1A2 gene is expressed in several tissues, including the liver, brain, eye, and proximal digestive tract [56] (Figure 2). It has been tested for association with MTX clearance in six studies [27,29,48,49,50,51] but the evidence for association is weak.

The two studies directly testing an association between the rs4149009 A allele and MTX concentration did not find an association [29,48] but one found that patients with <1 umol/L MTX at 42 h were enriched for the AA variant genotype compared to GA and GG patients, suggesting that the variant A allele is associated with increased MTX clearance. The GWAS found two SNPs in *SLCO1A2* reached genome-wide significance, but likely because they were in LD with SNPs in the neighboring *SLCO1B1* gene, not because they were functionally affecting SLCO1A2 [50].

##### SLCO1B1

The solute carrier organic anion transporter family member 1B1 gene is nearly exclusively expressed in the liver where it is located at the basolateral membrane of the hepatocytes [56] (Figure 2). Substrates of the SLCO1B1 (OATP1B1) transporter include endogenous molecules such as bilirubin and estrogens, as well as drugs, most notably statins and MTX. Genome-wide association studies identified variants in *SLCO1B1* as the only gene associated with MTX clearance at the usual genome-wide significance threshold [49,50]. In sequencing patients with the fastest and slowest MTX clearance, rare variants with large effects were identified, and the function was verified in vitro [51]. The SNP most often tested for association with MTX PK measures is rs4149056, which encodes a T521C transition that reduces the localization of the transporter to the surface of the cell and causes a severe reduction in transport of MTX in vitro [64]. This variant was universally associated with decreased MTX clearance among all the studies included in this review [21,22,27,28,32,33,34,35,38,43,49,50,51,52,54,55]. The rs4149056 common variant is included in two * alleles, the no function *5 (no other coding variants) allele and the more common *15 (with rs2306283 A > G) allele. Other decreased function alleles associated with reduced MTX clearance and reduced in vitro transport include *23 and *31 [51]. There were also alleles associated with increased MTX clearance [51] and transporter expression [65], *14 and *35. The rs2306283 A > G variant is included in many * alleles, and is associated with increased transporter expression. Most studies found that this SNP associated with faster MTX clearance [33,54], but one found that it associated with slower clearance [27].

##### SLCO1B3

The solute carrier organic anion transporter family member 1B3 gene is also exclusively expressed in the liver [56] (Figure 2) and is located on chromosome 12 next to *SLCO1B1.* In vitro studies have shown that it transports MTX [66]. None of the eleven tested variants in *SLCO1B3* influenced the 72 h MTX concentration in one study [29] or reached genome-wide significance in the GWAS, though two variants were close (*p* < 3 × 10^−6^) [49,50,51].

#### 3.3.2. Folate Pathway Genes

The following information is summarized and presented in Table 2.

##### ATIC

The 5-aminoimidazole-4-carboxamide ribonucleotide transformylase gene (ATIC) encodes a bifunctional enzyme that catalyzes the last two steps of the de novo purine pathway’s last two steps [56] (Figure 3). Due to its function as a pharmacodynamic enzyme and inclusion in MTXs mechanism of action, ATIC has been included as a candidate gene. However, three individual genome-wide association studies related to MTX clearance [49,50,51] did not find any genome-wide significance for ATIC. One study, in particular, on the polymorphism of ATIC 347 C > G allele concluded the variants had zero influence in higher 48 h concentrations [45].

##### ARID5B

*ARID5B*, encodes a member of the AT-rich interaction domain (ARID) family of DNA-binding proteins. The protein is widely expressed in most tissue types [56] and is associated with adipogenesis and liver development. It mainly serves as a transcriptional modulator and regulates targeted gene expression by recruiting PHD finger protein 2 [82]. This gene is also involved in cell growth and differentiation of B-lymphocyte progenitors, and is associated with the incident and relapse of ALL. However, underlying mechanisms by which *ARID5B* genotype impacts the susceptibility and treatment outcome remain uncertain. The current premise is that treatment outcome is related to the cancer cell’s sensitivity to antimetabolite drugs, including 6-mercaptopurine and MTX.

Five studies included SNPs in *ARID5B* [21,32,49,50,51] and only two of these studies found that SNPs reached the statistical threshold. One article [21] found a significant association linking rs4948496 with MTX concentrations, while the other study [32] was unable to find an association between rs4948496 and MTX concentration. This article [21] classified only the rs4948496 (T to C) variant, for which they found increased MTX concentrations at time points 24, 36 and 48 h. Both studies do suggest that the CC genotypes are responsible for decreased MTX clearance. This study also found significant PK associations between rs4948502 and increased serum concentration of 7-OH-MTX.

##### DHFR

Dihydrofolate reductase (DHFR) is an enzyme that converts dihydrofolate into tetrahydrofolate (Figure 3). The folic acid derivative is required for the de novo synthesis of purines, thymidylic acid, and several amino acids. The pharmacodynamic target of MTX is inhibition of DHFR, where the affinity of *DHFR* for MTX is 1000-fold higher than for folate. However, the GWAS studies [49,50,51] and PK studies [27,43,75] did not find evidence supporting DHFR polymorphisms to be associated with MTX PK.

##### GGH

Gamma-glutamyl hydrolase or GGH is a lysosomal peptidase that hydrolyzes gamma-linked polyglutamates on folate derivates, converting long-chain MTX polyglutamates (MTX-PGs) into a short-chain MTX-PGs and sequentially to MTX (Figure 3). Therefore, the modulation of *GGH* may affect the chemosensitivity of cancer cells, and exogenous folate levels may further modify this effect [56].

Four candidate gene studies examined five *GGH* polymorphisms (rs11545078, rs11545077, rs1800909, rs11545076, rs3758149) and MTX PK [20,67,68,83]. The rs3758149 variant (C > T) CT, and TT genotypes were specifically examined in two additional studies [20,67]. They focused on the variant due to its higher recurrence rate in patients with MTX serum levels >40 µmol at 24 h compared to CC wild type and proposed that the variant T allele was associated with decreased MTX clearance. Although no observable change in 48 h MTX concentration was found in two studies [20,68], it was significantly associated with MTX concentration in one [67].

Lastly, there is evidence that the methylation levels of CpG units for the *GGH* promoter region 14 affect MTX PK [83].

##### FPGS

Folylpolyglutamate synthetase (FPGS) is responsible for the conversion of MTX into MTXPGs (Figure 3). Loss of function leads to reduced intercellular concentration of MTXPG and results in MTX drug resistance [56]. The variants (rs1544105, rs10106, and rs4451422) were studied across four studies [21,67,69,70]. Three studies [67,69,70] reported that the rs1544105 (A to G) variant GG genotype resulted in a non-significant reduction in the 24 h MTX serum concentration compared to the AA and AG genotypes. One study [70] concluded that the rs1544105 genotype significantly influences the MTX concentration/dose values at 24 h after start of the high-dose MTX infusion.

##### MTHFD1

The trifunctional enzyme methylenetetrahydrofolate dehydrogenase (MTHFD1) is highly expressed in the liver [56]. The enzyme catalyzes tetrahydrofolate reduction in three sequential reactions of 10-formyltetrahydrofolate 5,10-methylenetetrahydrofolatedehydrogenase/5,10-methenyltetrahydrofolate-cyclohydrolase/10-formyltetrahydrofolate resulting in the analogous 10-formyl, 5,10-methenyl and 5,10-methylene derivatives (Figure 3). The three derivatives are all part of the one-carbon folate pool and required for biosynthetic processes, including purine and thymidine synthesis [84]. Four polymorphisms in this gene were investigated in six different studies [21,35,49,50,51,71]. One, rs2236225, was studied directly and associated with MTX PK but with inconsistent results among the included studies [21,35,71]. One of the studies noted an association between (GA and AA) variants and lowered 24 h MTX serum concentration, but the observation did not pass the significance threshold (MTX AUC) after correction for multiple comparisons [35]. The c.1958G > A, rs2236225 variant in MTHFD1 has been associated with reduced histological response [85], but no significant effects were observed in our study.

##### MTHFR

The enzyme methylenetetrahydrofolate reductase (MTHFR) is responsible for the conversion of 5,10-methylenetetrahydrofolate to 5-methyltetrahydrofolate which is needed for the transformation of the toxic homocysteine to methionine (Figure 3). The enzyme is expressed in most human cells but it is not directly involved in the transport of MTX [56]. It has been hypothesized that reduced expression of *MTHFR* would be associated with MTX-induced toxicity in patients with leukemia but clinical studies show conflicting results on this matter [86]. The T allele in SNP rs1801133 causes a change of alanine to valine in the protein, resulting in decreased enzyme activity of *MTHFR*. Seven of 28 candidate gene studies found that the T allele in rs1801133 was associated with decreased elimination of MTX whereas no statistically significant association was found in 20 other studies [23,25,26,27,28,32,33,34,35,39,42,43,45,46,47,52,57,67,71,72,73,74,75,76,77,78,79,80]. The second most studied SNP, rs1801131, was included in 17 candidate gene studies but none of these studies found a clinically significant association between MTX elimination and this SNP.

##### MTR

5-methyltetrahydrofolate (5-methyl-THF) is a vitamin B12-dependent enzyme also involved in the folate-mediated one-carbon metabolism. It catalyzes the methylation of homocysteine to methionine with the simultaneous conversion of 5-methyl-tetrahydrofolate (5-methyl-THF) to tetrahydrofolate (THF) [56] (Figure 3). The variant rs1805087 within MTR has previously been ascribed to toxicity outcomes. However, none of the reported GWAS studies were able to establish an association between *MTR* and MTX PK. Several candidate gene studies investigated rs1805087 for PK or toxicity measures and failed to find any observable differences in concentration or AUC.

##### MTRR

5- methyltetrahydrofolate-homocysteine methyltransferase reductase (MTRR) is primarily involved in the reductive methylation of homocysteine to methionine, utilizing methylcobalamin as an intermediate methyl carrier [56] (Figure 3). Seven variants in *MTRR* were examined. One particular variant, rs1801394, was tested in four separate candidate gene studies [21,28,35,57]. However, only one of the four studies [28] found that the rs1801394 (AG and GG) variants were associated with significantly lower 24 h MTX concentrations as opposed to AA wild types. The study found no difference at the 48 h MTX concentration, suggesting that variant rs1801394 G allele remains associated with increased MTX clearance.

##### TYMS

The enzyme thymidylate synthase (TYMS) is essential for the de novo production of purines in the DNA synthesis [87]. MTXPG inhibits the enzyme and thereby prevents the transfer of one-carbon from 5,10-methylenetetrahydrofolate to deoxyuridine monophosphate (dUMP) in the formation of deoxythymidine monophosphate (dTMP) (Figure 3). Low expression of *TYMS* in lymphoblastic leukemia cells is associated with decreased antileukemic effects of MTX and increased risk of relapse [88]. *TYMS* is ubiquitously expressed in normal human tissue, and thought to be important for basal cellular functions but not directly involved in the transport of MTX [56]. The role of the enzyme in relation to MTX-induced toxicity was examined in several candidate gene studies, which simultaneously searched for associations with MTX PK [25,33,34,42,43,46,57,71]. The most common studied SNPs was rs34743033, known as the tandem repeat [25,33,34,42,43,46,57,71]. The combination of increased *TYMS* expression (tandem repeat x3) and reduced function of *MTHFR* (homozygous for the 677T allele) was associated with increased risk of MTX-induced hematologic toxicity but not plasma MTX concentrations [71]. Only two out of 14 studies found an association between SNPs in *TYMS* and MTX elimination [42,81].

#### 3.3.3. Additional Genes Examined

Several additional genes were sparsely examined and do not fit into neither the folate pathway nor transporter gene categories. *ELMO1* [89], *NALCN* [89], *NRII2* (SXR) [20], *SULT1E1* [90], *UGT1A1* [46], and four intergenic polymorphisms [89] (rs3862227, rs4888024, rs4905865, and rs1359645) were all found to significantly affect MTX PK. While significant, these genes have only been reported once each. Replication would be needed in order to definitively state that these genes do affect MTX PK. *CCND1*, *CYP3A4*, *CYP3A5*, *GSTM1*, *GSTP1*, *GSTT1*, *JAK1*, *JAK3*, *TPMT*, *TSG1*, *SHMT1*, and *VDR* all failed to significantly affect MTX PK, though rationale for their selection was often indirect of MTX PK.

## 4. Discussion

The large inter-individual variation in MTX clearance can only in part be explained by differences in clinical patient characteristics and cancer treatment protocols. In this systematic review, we evaluated 58 articles that studied the association between SNPs and PK variables in relation to infusions with high-dose MTX. Most of these candidate gene studies looked at the association between MTX PK parameters and common SNPs in genes encoding transporter proteins (Table 1, Figure 2) or folate pathway genes (Table 2, Figure 3), whereas rare non-synonymous mutations were less often included. The only gene with variants influencing MTX PK was *SLCO1B1*, which is a bit surprising as it is expressed in the liver, and the majority of MTX is eliminated through the kidney.

During and after a high-dose MTX infusion, the majority of MTX is eliminated through the kidney, although enterohepatic circulation plays a substantial role. In vitro studies have shown that several renal and hepatic transporter proteins have affinity for MTX, and PK studies in knock-out mice have suggested that these transporters are involved in the distribution and elimination of MTX [63,91,92]. Furthermore, cases of patients with severely delayed MTX elimination have contributed to the idea that transporter proteins and enzymes in the folate pathway can affect both MTX clearance and toxicity [93,94]. During a high-dose MTX infusion, the plasma concentration of MTX is probably so high that most of the drug is eliminated by glomerular filtration. Hepatic uptake of MTX probably affects the initial distribution of the drug, whereas active transport mediated renal transporter proteins could play a more important role in eliminating MTX at low plasma MTX concentrations.

Variants in the *SLCO1B1* gene have been associated with increased clearance as well as decreased clearance, and the phenotypes are well defined by the Clinical Pharmacogenetics Implementation Consortium because it is the subject of a guideline for simvastatin. As of this writing, the PharmVar consortium is working on renaming and consolidating alleles, and will publish these in the future [95]. The evidence is building for utilizing *SLCO1B1* in dosing recommendations for MTX, though there is still a lot of variability between courses of high-dose MTX that is not accounted for by these variants. One study showed variants in this gene accounted for >10% of the between-subject variability, which was more than clinical variables. Rare *SLCO1B1* SNPs that negatively influence the cellular uptake of MTX were also associated with low MTX clearance after infusions with high-dose MTX [51]. A possible translation of this information into clinical changes could be that patients with important SNPs in *SLCO1B1* should receive closer monitoring with early measurement of plasma MTX concentrations in order to optimize the supportive care (*e.g*., increased hydration or urine alkalization) in case of decreased MTX clearance.

The other genes most studied were *MTHFR* and *SLC19A1*, but neither had a majority of studies reporting significant associations. Overall, *SLC19A1* is an important transporter responsible for folate homeostasis. Folate is a crucial nutrient that supports important physiological functions such as purine and pyrimidine biosynthesis and regulates the production of one-carbon donors for DNA methylation. Evidence suggests that polymorphisms in *SLC19A1* increase the susceptibility to birth defects, which is why prenatal folate supplementation is highly recommended. Mice lacking the *Slc19a1* gene die in utero [96]. From an evolutionary perspective, damaging polymorphisms in SLC19A1 could have been selected against to reduce the occurrence these abnormalities. As such, persisting polymorphisms in *SLC19A1* may occur, but their effect on the transport functionality of *SLC19A1* may be minimal. People with Down Syndrome have a third copy of *SLC19A1*, since it is on chromosome 21. Patients with Down Syndrome treated with high-dose MTX are very sensitive to toxicities and usually receive reduced doses, indicating that perhaps expression but not genetic variants are associated with MTX-induced toxicities. *MTHFR* plays a central role in folate metabolism by catalyzing the conversion of 5,10-methylenetetrahydrofolate (THF) to 5-methylTHF. This is the primary circulating form of folate, which is needed to reduce the toxic homocysteine to methionine. Through this process, folate is an important donor of methyl-groups for all intracellular methylation processes. *MTHFR*—though an important enzyme in intracellular folate metabolism—is not directly involved in MTX transport or in the direct process of purine and pyrimidine synthesis. Based on the limited significant findings and high volume of conflicting evidence, it is unlikely that polymorphisms in *SLC19A1* and *MTHFR* significantly affect high-dose MTX PK in pediatric patients with ALL, osteosarcoma, or lymphoma.

### 4.1. Toxicity

A decrease in MTX clearance, and thereby elevated MTX tissue exposure, potentially leads to an increased risk of MTX-induced toxicity, although patients with elevated plasma MTX concentrations receive additional folinic acid to rescue the normal tissue, which may confound associations between MTX PK and toxicity. Furthermore, decreased function of MTX transporter proteins or enzymes in the folate pathway can affect the intracellular handling of MTX independently of plasma MTX levels and thereby lead to increased toxicity despite normal systemic MTX clearance. Patients developing severe nephrotoxicity are at risk of life-threatening MTX exposure requiring treatment with glucarpidase as a means to enzymatically eliminate the drug. SNPs in *SLCO1B1*, *MTHFR* and *SLC19A1* have been studied extensively—with or without PK data—in relation to MTX-induced toxicity, mostly in the pediatric ALL population. Only *SLCO1B1* SNPs have been associated with MTX PK and toxicity in a genome-wide association studies. *SLCO1B1* rs4149056 (TT or TC genotype) and rs11045879 (CC and TC genotype) have been quite consistently associated with decreased clearance and elevated MTX levels in combination with a lower frequency of gastrointestinal toxicity and in a few cases with increased nephrotoxicity and hepatotoxicity [25,29,33,38,49]. The association of *MTHFR* and *SLC19A1* SNPs with toxicity is less clear. *MTHFR* rs1801133 (C677T–CT or TT genotype) has been associated with increased MTX levels and decreased MTX clearance [23,26,39,57,71,72,76,78]. However, an equal amount of studies have failed to show this association [28,32,34,35,45,46,47,52,73,74,75,77,80]. In one study, even when there was an association with higher MTX levels, a genetic association of this SNP with renal toxicity could not be established suggesting that other clinical and genetic factors play a role [23]. The most frequent toxicities associated with *MTHFR* rs1801133 were mucositis and myelosuppression [57,72,73,74]. The role of *SLC19A1* rs1051266/rs61510559 (G80A) in toxicity remains unclear. Many studies have reported no association between this SNP and MTX PK [20,22,23,28,38,45] and others have shown contradicting results showing both the AA as the GG variant associated with increased clearance [29,39,40,41]. *SLC19A1* rs1051266/rs61510559 (G80A) has been associated with gastrointestinal toxicity and hepatotoxicity [21,47]. Overall, data on MTX pharmacogenetics in relation to toxicity have shown very inconsistent results and even when results were significant, effect sizes were often small. This is often observed in pharmacogenomic studies of complex traits such as the multifactorial process of developing MTX toxicity [97,98]. The transporter and enzyme pathways of MTX are complex with many escape mechanisms. This and the fact that patients differ in clinical characteristics hampers finding one single pharmacogenetic variant to be associated with toxicity. It stresses the fact that PK measures, such as clearance and MTX levels, might be more effective in predicting toxicity than single pharmacogenetic variants.

### 4.2. Limitations and Future Studies

In articles included in this review, most common SNPs in genes encoding transporter proteins and enzymes in the folate pathway explain little or none of the inter-individual variation in MTX elimination after infusions with high-dose MTX. Most of the studies included were candidate gene studies, but there were three genome-wide association studies. Genome-wide association studies rely on testing mostly intronic SNPs that are in linkage disequilibrium (LD) with variants that affect function. LD is the association among nearby DNA variations such that the alleles at neighboring polymorphisms are connected within a population more frequently than if they were unlinked, and one needs to only test one variant in the LD block to be fairly certain about the rest in the block. The statistical power of genome-wide association studies and the ability to identify an association between variation and phenotype depends on several factors, including the frequency of the minor allele or genotype, the relevant risk given by the disease-associated allele or genotype, the relationship between the genotyped marker and the minor allele, size of the study, disease predominance, and genetic diversity in the population cohort [99]. Consequently, the method is limited to its ability to detect genetic association by LD. Most of the studies in this review included less than 200 patients and as such did not have the power to detect the impact of rare SNPs on MTX clearance or toxicity. Therefore, it would be beneficial for future studies to investigate if rare SNPs significantly alter the function of transporter proteins or enzymes in patients with severely delayed MTX elimination or unexpected intense toxicity. Furthermore, future studies should consider replicating and validating the select few genes with variants that significantly affected MTX exposure. In recent years, machine learning (ML) has been an emerging branch of computational methods that can enhance genome-wide association studies performance and interpretation and identify new SNP associations. ML uses algorithms to build mathematical models acquired from training on data and learning patterns. By combining data sources with genotype, ML data are uniquely positioned to discover the hidden biological interactions for better prediction and diagnosis of complex diseases [100].

## 5. Conclusions

In conclusion, in this extensive literature review, the only gene with the majority of studies concluding that a variant influences MTX PK was *SLCO1B1*. A possible clinical implication could be that patients with mutations in the *SLCO1B1* gene putting them at risk of decreased MTX clearance should receive closer monitoring with early measurement of plasma MTX concentrations in order to optimize supportive care. Current studies are often underpowered and unfit to study rare phenotypes with severely delayed MTX clearance and rare non-synonymous mutations in relation to MTX PK parameters. Sequencing outliers with severely delayed clearance may identify novel variants that have not been discovered by genotyping previously. For future studies, collaboration to increase power is essential.

## Figures and Tables

**Table 1:** Transporter Genes That Significantly Affect Methotrexate Exposure.

Gene Name	Variants	Effect of Variant on Methotrexate Exposure	References
*ABCB1*	rs9282564	↑	[20,^21^]
*ABCG2*	rs12505410	↓	[22]
*ABCG2*	rs13120400	↑	[22]
*ABCG2*	rs13137622	↓	[22]
*ABCG2*	rs2231142	↑	[20,^23^,^24^,^25^,^26^,^27^]
*ABCC2*	rs3740065	↑ *|↓	[22,28,29*,^30^*,^31^*]
*ABCC2*	rs3740066	↑ *|↓	[20,^27^*,^29^*]
*ABCC2*	rs717620	↑ *|↓	[20,22,23,27,28,29,30*,31*,32,^33^,^34^,^35^,^36^]
*ABCC3*	rs4793665	↓	[20,^36^]
*ABCC3*	rs9895420	↓	[36]
*ABCC4*	rs10219913	↑	[28,^29^]
*ABCC4*	rs7317112	↑	[28,^29^]
*ABCC4*	rs868853	↓	[29,31,37]
*ABCC4*	rs9516519	↑ *|↓	[28*,29,^31^*]
*SLC19A1*	rs1051266	↑ *|↓	[20,21,22,26*,^27^,^28^,^29^*,^33^,^34^,^35^,^38^,^39^,40*,41*,42*,43*]
*SLC19A1*	rs1051296	↑	[44]
*SLC19A1*	rs61510559	↓	[23,25,45,^46^,^47^]
*SLCO1A2*	rs4149009	↓	[27,29,48]
*SLCO1B1*	rs10841753	↑ *|↓	[38,49*,50]
*SLCO1B1*	rs11045787	↑	[49]
*SLCO1B1*	rs11045818	↑ *|↓	[21,49*,50*]
*SLCO1B1*	rs11045813	↑	[50]
*SLCO1B1*	rs11045819	↓	[21,51]
*SLCO1B1*	rs11045821	↓	[50]
*SLCO1B1*	rs11045825	↓	[50]
*SLCO1B1*	rs11045870	↓	[50]
*SLCO1B1*	rs11045872	↓	[22,49,50]
*SLCO1B1*	rs11045879	↑ *|↓	[22,25*,29,^33^,35*,49,50*,^52^]
*SLCO1B1*	rs11045892	↓	[50]
*SLCO1B1*	rs11045897	↑	[38,53]
*SLCO1B1*	rs16923647	↓	[50]
*SLCO1B1*	rs17328763	↑	[49]
*SLCO1B1*	rs2169969	↓	[50]
*SLCO1B1*	rs2306283	↑ *|↓	[27*,33,^38^,54]
*SLCO1B1*	rs2900476	↓	[22,49]
*SLCO1B1*	rs34671512	↓	[51]
*SLCO1B1*	rs4149056	↑	[21,^22^,^27^,28,^32^,33,^34^,35,^38^,^43^,49,50,51,^52^,54,55]
*SLCO1B1*	rs4149076	↓	[49]
*SLCO1B1*	rs4149081	↑ *|↓	[22*,49,50*,25*,53*]
*SLCO1B1*	rs59502379	↑	[51]

Genes and variants not found in the table above do not significantly affect methotrexate exposure. Please see Table S2 in Supplementary for full list of genes and variants. Underlined references found a significant association between variant and methotrexate exposure. * Indicates the studies that showed increased MTX exposure when there were conflicting reports for a variant. ↑ Indicates that the variant is associated with increased/higher methotrexate exposure. ↓ Indicates that the variant is associated with decreased/lower methotrexate exposure.

**Table 2:** Folate Pathway Genes That Significantly Affect Methotrexate Exposure.

Gene Name	Variants	Effect of Variant on Methotrexate Exposure	References
*ARID5B*	rs4948496	↑	[21,^32^]
*GGH*	rs3758149	↑	[20,67,^68^]
*FPGS*	rs1544105	↓	[69,70,^67^,^21^]
*MTHFD1*	rs2236225	↓	[21,35,^71^]
*MTHFR*	rs1801133	↑|↓ *	[23,^25^,26,^27^,^28^,^32^,^33^,^34^,^35^,39,42*,^43^,^45^,^46^,^47^,^52^,57,^67^,71*,72,^73^,^74^,^75^,^76^,^77^,78,^79^,^80^]
*TYMS*	rs2790	↓	[81]
*TYMS*	rs34743033	↓	[25,33,34,42,^43^,^46^,^57^,^71^]

Genes and variants not found in the table above do not significantly affect methotrexate exposure. Please see Table S2 in Supplementary for full list of genes and variants. Underlined references found a significant association between variant and methotrexate exposure. * Indicates the studies that showed decreased MTX exposure when there were conflicting reports for a variant. ↑ Indicates that the variant is associated with increased/higher methotrexate exposure. ↓ Indicates that the variant is associated with decreased/lower methotrexate exposure.

## Data Availability

All the data used for this study are available in Table S2. The search strategy can be replicated with the terms found in Table S1.

## Supplementary Materials

The following are available online at https://www.mdpi.com/article/10.3390/cancers13112837/s1. Table S1: Search query used for PubMed and Scopus for article identification; Table S2: All available genes and polymorphisms included in the analysis for this systematic literature review.
