# Supplementary material for: Systematic Review of Pharmacogenetic Factors That Influence High-Dose Methotrexate Pharmacokinetics in Pediatric Malignancies
Source: Cancers (Basel). 2021 Jun 7;13(11):2837. doi: 10.3390/cancers13112837 (PMC8201112; doi:10.3390/cancers13112837)
Supplement: Supplementary file 1 [file cancers-13-02837-s001.zip › cancers-1223411-supplementary.pdf]

Supplement to:

Systematic Review of Pharmacogenetic Factors that Influence High-Dose Methotrexate Pharmacokinetics in Pediatric Malignancies

Zachary L. Taylor[1,2,3], Jesper Vang[4], Elixabet Lopez-Lopez[5,6], Natanja Oosterom[7], Torben Mikkelsen[8], Laura B. Ramsey[2,3,*]

[1] Department of Pharmacology and Systems Physiology, University of Cincinnati, Cincinnati, OH, 45267, USA; taylorzl@mail.uc.edu
[2] Division of Research in Patient Services, Cincinnati Children's Hospital Medical Center, Cincinnati, OH, 45229, USA
[3] Division of Clinical Pharmacology, Cincinnati Children's Hospital Medical Center, Cincinnati, OH, 45229, USA
[4] Department of Health Technology, Technical University of Denmark, Lyngby 2800, Denmark; Paediatric Oncology Research Laboratory, University Hospital of Copenhagen, Rigshospitalet Blegdamsvej 9, 2100 Copenhagen, Denmark; jvan@dtu.dk
[5] Department of Genetics, Physical Anthropology and Animal Physiology, Faculty of Science and Technology, University of the Basque Country, UPV/EHU, Leioa, 48940, Spain; elixabet.lopez@ehu.eus
[6] Pediatric Oncology Group, BioCruces Bizkaia Health Research Institute, Barakaldo, 48903, Spain
[7] Princess Máxima Center for Pediatric Oncology, Utrecht, 3720, The Netherlands; n.oosterom@prinsesmaximacentrum.nl
[8] Department of Pediatric Oncology, Aarhus University Hospital, Aarhus, 8200, Denmark; torben.mikkelsen@clin.au.dk
* Correspondence: laura.ramsey@cchmc.org; Tel.: +1-513-803-8963

Supplementary Materials: Table S1: Search query used by PubMed and Scopus for article identification.

Database	Search Query	Number of Hits	Date of Search
PubMed	(("methotrexate"[MeSH Terms] OR "methotrexate"[Text Word]) NOT "review"[Publication Type]) AND "english"[Language] AND (((("enzym*"[Text Word] OR "enzymes"[MeSH Terms] OR "enzymes and coenzymes"[MeSH Terms] OR "transport*"[Text Word] OR "membrane transport proteins"[MeSH Terms] OR "gene*"[Text Word] OR "genes"[MeSH Terms] OR "renal*"[Text Word] OR "kidney*"[Text Word] OR "kidney"[MeSH Terms] OR "hepatic*"[Text Word] OR "liver"[Text Word] OR "liver"[MeSH Terms]) AND ("pharmacogen*"[Text Word] OR "pharmacogenetics"[MeSH Terms] OR "genetic variation"[MeSH Terms] OR "genetic*"[Text Word] OR "genetics, medical"[MeSH Terms] OR "genotype"[MeSH Terms] OR "mutation"[MeSH Terms] OR "mutation"[Text Word] OR "pharmacogenomic variants"[MeSH Terms] OR "polymorphism, genetic"[MeSH Terms] OR "polymorphism, single nucleotide"[MeSH Terms] OR "polymorphism*"[Text Word] OR "varia*"[Text Word])) NOT "review"[Publication Type]) AND "english"[Language]) AND ((("pharmacokinetic*"[Text Word] OR "pharmacokinetics"[MeSH Terms] OR "clearance"[Text Word] OR "elimination"[Text Word] OR "metabolic clearance rate"[MeSH Terms] OR "exposure"[Text Word] OR "area under curve"[MeSH Terms] OR "area under curve"[Text Word] OR "concentration*"[Text Word] OR "concentration-time"[Text Word] OR "plasma"[Text Word] OR "metabol*"[Text Word] OR "precision medicine"[Text Word] OR "precision medicine"[MeSH Terms] OR "modeling"[Text Word] OR "simulation"[Text Word] OR "personalized medicine"[Text Word]) NOT "review"[Publication Type]) AND "english"[Language]) AND ((("pediatric*"[Text Word] OR "pediatrics"[MeSH Terms] OR "child*"[Text Word] OR "child"[MeSH Terms] OR "adolesc*"[Text Word] OR "adolescent"[MeSH Terms] OR "paediatric*"[Text Word] OR "infant*"[Text Word] OR "infant"[MeSH Terms] OR "teen*"[Text Word]) NOT "review"[Publication Type]) AND "english"[Language])	437	11/16/2020

PubMed	(("methotrexate"[MeSH Terms] OR "methotrexate"[Text Word]) NOT "review"[Publication Type]) AND "english"[Language] AND (((("enzym*"[Text Word] OR "enzymes"[MeSH Terms] OR "enzymes and coenzymes"[MeSH Terms] OR "transport*"[Text Word] OR "membrane transport proteins"[MeSH Terms] OR "gene*"[Text Word] OR "genes"[MeSH Terms] OR "renal*"[Text Word] OR "kidney*"[Text Word] OR "kidney"[MeSH Terms] OR "hepatic*"[Text Word] OR "liver"[Text Word] OR "liver"[MeSH Terms]) AND ("pharmacogen*"[Text Word] OR "pharmacogenetics"[MeSH Terms] OR "genetic variation"[MeSH Terms] OR "genetic*"[Text Word] OR "genetics, medical"[MeSH Terms] OR "genotype"[MeSH Terms] OR "mutation"[MeSH Terms] OR "mutation"[Text Word] OR "pharmacogenomic variants"[MeSH Terms] OR "polymorphism, genetic"[MeSH Terms] OR "polymorphism, single nucleotide"[MeSH Terms] OR "polymorphism*"[Text Word] OR "varia*"[Text Word])) NOT "review"[Publication Type]) AND "english"[Language]) AND ((("pharmacokinetic*"[Text Word] OR "pharmacokinetics"[MeSH Terms] OR "clearance"[Text Word] OR "elimination"[Text Word] OR "metabolic clearance rate"[MeSH Terms] OR "exposure"[Text Word] OR "area under curve"[MeSH Terms] OR "area under curve"[Text Word] OR "concentration*"[Text Word] OR "concentration-time"[Text Word] OR "plasma"[Text Word] OR "metabol*"[Text Word] OR "precision medicine"[Text Word] OR "precision medicine"[MeSH Terms] OR "modeling"[Text Word] OR "simulation"[Text Word] OR "personalized medicine"[Text Word]) NOT "review"[Publication Type]) AND "english"[Language]) AND ((("pediatric*"[Text Word] OR "pediatrics"[MeSH Terms] OR "child*"[Text Word] OR "child"[MeSH Terms] OR "adolesc*"[Text Word] OR "adolescent"[MeSH Terms] OR "paediatric*"[Text Word] OR "infant*"[Text Word] OR "infant"[MeSH Terms] OR "teen*"[Text Word]) NOT "review"[Publication Type]) AND "english"[Language])	443	2/26/2021
Scopus	((TITLE-ABS-KEY(methotrexate)) AND ((TITLE-ABS-KEY(enzym*) OR TITLE-ABS-KEY(transport*) OR TITLE-ABS-KEY(kidney*) OR TITLE-ABS-KEY(renal*) OR TITLE-ABS-KEY(liver) OR TITLE-ABS-KEY(hepatic*)) AND (TITLE-ABS-KEY(pharmacogen*) OR TITLE-ABS-KEY(polymorphism) OR TITLE-ABS-KEY(mutation) OR TITLE-ABS-KEY(varia*) OR TITLE-ABS-KEY(gene*) OR TITLE-ABS-KEY(geno*))) AND (TITLE-ABS-KEY(clearance) OR TITLE-ABS-KEY(concentration) OR TITLE-ABS-KEY(elimination) OR TITLE-ABS-KEY(exposure) OR TITLE-ABS-KEY(metabo*) OR TITLE-ABS-KEY(pharmacokinetic*) OR TITLE-ABS-	486	11/16/2020

	KEY(modeling) OR TITLE-ABS-KEY(simulation) OR TITLE-ABS-KEY(parameter*)) AND (TITLE-ABS-KEY(pediatric*) OR TITLE-ABS-KEY(child*) OR TITLE-ABS-KEY(adolesc*) OR TITLE-ABS-KEY(paediatric*) OR TITLE-ABS-KEY(teen*) OR TITLE-ABS-KEY(infant*)) AND (TITLE-ABS-KEY(cancer) OR TITLE-ABS-KEY(leukemia) OR TITLE-ABS-KEY(lymphoma) OR TITLE-ABS-KEY(osteosarcoma) OR TITLE-ABS-KEY(oncology) OR TITLE-ABS-KEY(neoplasm*)) AND NOT DOCTYPE(re) AND LANGUAGE(english))		
Scopus	((TITLE-ABS-KEY(methotrexate)) AND ((TITLE-ABS-KEY(enzym*) OR TITLE-ABS-KEY(transport*) OR TITLE-ABS-KEY(kidney*) OR TITLE-ABS-KEY(renal*) OR TITLE-ABS-KEY(liver) OR TITLE-ABS-KEY(hepatic*)) AND (TITLE-ABS-KEY(pharmacogen*) OR TITLE-ABS-KEY(polymorphism) OR TITLE-ABS-KEY(mutation) OR TITLE-ABS-KEY(varia*) OR TITLE-ABS-KEY(gene*) OR TITLE-ABS-KEY(geno*))) AND (TITLE-ABS-KEY(clearance) OR TITLE-ABS-KEY(concentration) OR TITLE-ABS-KEY(elimination) OR TITLE-ABS-KEY(exposure) OR TITLE-ABS-KEY(metabo*) OR TITLE-ABS-KEY(pharmacokinetic*) OR TITLE-ABS-KEY(modeling) OR TITLE-ABS-KEY(simulation) OR TITLE-ABS-KEY(parameter*)) AND (TITLE-ABS-KEY(pediatric*) OR TITLE-ABS-KEY(child*) OR TITLE-ABS-KEY(adolesc*) OR TITLE-ABS-KEY(paediatric*) OR TITLE-ABS-KEY(teen*) OR TITLE-ABS-KEY(infant*)) AND (TITLE-ABS-KEY(cancer) OR TITLE-ABS-KEY(leukemia) OR TITLE-ABS-KEY(lymphoma) OR TITLE-ABS-KEY(osteosarcoma) OR TITLE-ABS-KEY(oncology) OR TITLE-ABS-KEY(neoplasm*)) AND NOT DOCTYPE(re) AND LANGUAGE(english))	492	2/26/2021

Table S2: All available genes and polymorphisms included in the analysis for this systematic literature review.

Gene Name	Variants	Significant Findings?	Does variant increase exposure?	References
ABCB1	rs10244266	No	NA	29
ABCB1	rs10246878	No	NA	29
ABCB1	rs10260862	No	NA	29
ABCB1	rs10264990	No	NA	22, 29
ABCB1	rs10267099	No	NA	22
ABCB1	rs10268314	No	NA	29
ABCB1	rs10274587	No	NA	29
ABCB1	rs10276036	No	NA	22
ABCB1	rs10280101	No	NA	21, 22

ABCB1	rs1045642	No	Yes	20, 25, 26, 27, 34, 35, 38, 57
ABCB1	rs1055302	No	NA	29
ABCB1	rs10808071	No	NA	22
ABCB1	rs1128503	No	No	20, 21, 22, 27, 34, 38
ABCB1	rs1202171	No	NA	22, 29
ABCB1	rs1202172	No	NA	29
ABCB1	rs1202179	No	NA	21, 29
ABCB1	rs12535512	No	NA	29
ABCB1	rs13226726	No	NA	29
ABCB1	rs13229143	No	NA	29
ABCB1	rs13233308	No	NA	22, 29
ABCB1	rs17327624	No	NA	29
ABCB1	rs1922240	No	NA	29
ABCB1	rs2032582	No	Yes	21, 57
ABCB1	rs2214102	No	NA	29
ABCB1	rs2235013	No	NA	21, 29
ABCB1	rs2235035	No	NA	22
ABCB1	rs2235046	No	NA	29
ABCB1	rs2235048	No	NA	22, 29
ABCB1	rs2520464	No	NA	29
ABCB1	rs3789243	No	NA	22, 29
ABCB1	rs3842	No	NA	29
ABCB1	rs4148733	No	NA	29
ABCB1	rs4148734	No	NA	29
ABCB1	rs4148737	No	NA	22
ABCB1	rs4148738	No	NA	29
ABCB1	rs4148743	No	NA	29
ABCB1	rs6946119	No	NA	29
ABCB1	rs6961419	No	NA	29
ABCB1	rs6961649	No	NA	22
ABCB1	rs6961665	No	NA	22
ABCB1	rs6979885	No	NA	29
ABCB1	rs7789645	No	NA	29
ABCB1	rs9282564	**Yes**	Yes	20, 21
ABCC1	rs11075291	No	NA	22
ABCC1	rs12922588	No	NA	20
ABCC1	rs2074087	No	NA	27
ABCC1	rs215060	No	NA	20
ABCC1	rs2230671	No	No	27, 29
ABCC1	rs246219	No	NA	20, 22
ABCC1	rs246221	No	NA	20, 29

ABCC1	rs246240	No	NA	29
ABCC1	rs35592	No	NA	22, 29
ABCC1	rs3784862	No	NA	22, 27, 29
ABCC1	rs4148330	No	NA	20, 29
ABCC1	rs4148358	No	NA	20
ABCC10	rs1214748	No	NA	20
ABCC10	rs1214752	No	NA	20
ABCC10	rs831314	No	NA	20
ABCC2	rs12826	No	Yes* ǀ No	28, 29*
ABCC2	rs17222723	No	NA	36
ABCC2	rs1885301	No	NA	36
ABCC2	rs2002042	No	NA	22
ABCC2	rs2273697	No	No	20, 22, 27, 30, 35, 36
ABCC2	rs2756109	No	NA	22
ABCC2	rs2804402	No	NA	36
ABCC2	rs3740063	No	NA	22
ABCC2	rs3740065	**Yes**	Yes* ǀ No	22, 28, 29*, 30*, 31*
ABCC2	rs3740066	**Yes**	Yes* ǀ No	20, 27*, 29*
ABCC2	rs3740074	No	NA	22
ABCC2	rs4148398	No	NA	22
ABCC2	rs717620	**Yes**	Yes* ǀ No	20, 22, 23, 27, 28, 29, 30*, 31*, 32, 33, 34, 35, 36
ABCC2	rs7910642	No	NA	36
ABCC2	rs8187710	No	NA	36
ABCC3	rs10153257	No	NA	29
ABCC3	rs1051640	No	NA	29
ABCC3	rs11568591	No	NA	36
ABCC3	rs12051822	No	NA	29
ABCC3	rs12602161	No	NA	20, 29
ABCC3	rs12604031	No	NA	29
ABCC3	rs1541392	No	NA	29
ABCC3	rs1558288	No	NA	29
ABCC3	rs17562467	No	NA	29
ABCC3	rs17562516	No	NA	29
ABCC3	rs17563146	No	NA	29
ABCC3	rs1978153	No	NA	29
ABCC3	rs1989983	No	NA	36
ABCC3	rs2072365	No	NA	29
ABCC3	rs2107441	No	NA	20
ABCC3	rs2189595	No	NA	29
ABCC3	rs2277624	No	NA	29

ABCC3	rs2412332	No	NA	29
ABCC3	rs2412333	No	NA	20, 29
ABCC3	rs3785911	No	NA	29
ABCC3	rs3785912	No	NA	29
ABCC3	rs4148411	No	NA	29
ABCC3	rs4148412	No	NA	29
ABCC3	rs4148413	No	NA	29
ABCC3	rs4148416	No	NA	22
ABCC3	rs4148418	No	NA	29
ABCC3	rs4793665	**Yes**	No	20, 36
ABCC3	rs4793666	No	NA	29
ABCC3	rs7212045	No	NA	29
ABCC3	rs733392	No	NA	20, 29
ABCC3	rs739921	No	NA	29
ABCC3	rs739923	No	NA	29
ABCC3	rs757421	No	NA	29
ABCC3	rs8073706	No	NA	29
ABCC3	rs8075406	No	NA	29
ABCC3	rs8196	No	NA	29
ABCC3	rs879459	No	NA	29
ABCC3	rs9895420	**Yes**	No	36
ABCC4	rs10219913	**Yes**	Yes	28, 29
ABCC4	rs11568658	No	NA	37
ABCC4	rs1678392	No	No	28, 29
ABCC4	rs1764419	No	NA	37
ABCC4	rs2274407	No	Yes	27, 31, 37
ABCC4	rs2619312	No	No	28, 29
ABCC4	rs2993579	No	NA	37
ABCC4	rs3742106	No	NA	29, 37
ABCC4	rs3751333	No	NA	37
ABCC4	rs7317112	**Yes**	Yes	28, 29
ABCC4	rs868853	**Yes**	No	29, 31, 37
ABCC4	rs869951	No	NA	37
ABCC4	rs9302061	No	No	28, 29
ABCC4	rs9516519	**Yes**	Yes* | No	28*, 29, 31
ABCC5	rs1520195	No	NA	36
ABCC5	rs562	No	NA	36
ABCC5	rs7627754	No	NA	36
ABCG2	rs12505410	**Yes**	No	22
ABCG2	rs13120400	**Yes**	Yes	22
ABCG2	rs13137622	**Yes**	No	22

ABCG2	rs1564481	No	NA	22
ABCG2	rs17731538	No	NA	22
ABCG2	rs2231137	No	Yes	31, 38
ABCG2	rs2231142	**Yes**	Yes	20, 23, 24, 25, 26, 27
ABCG2	rs2622604	No	NA	22
ABCG2	rs2622621	No	Yes	22, 29
ABCG2	rs2725252	No	No	22, 29
ABCG2	rs3114018	No	NA	22
ABCG2	rs6857600	No	NA	22
ARID5B	rs10821936	No	NA	21
ARID5B	rs4506592	No	NA	21
ARID5B	rs4509706	No	NA	21
ARID5B	rs4948487	No	NA	21
ARID5B	rs4948496	**Yes**	Yes	21, 32
ARID5B	rs4948502	No	NA	21
ARID5B	rs7089424	No	NA	21
ATIC	rs2372536	No	NA	45
DHFR	rs1105525	No	NA	27
DHFR	rs1643641	No	NA	43
DHFR	rs1650695	No	NA	43
DHFR	rs1650696	No	NA	43
DHFR	rs1650697	No	NA	27
DHFR	rs408626	No	NA	43
DHFR	rs442767	No	NA	43
DHFR	rs70991108	No	NA	75
DHFR	rs7387	No	NA	27
FPGS	rs10106	No	NA	21
FPGS	rs1544105	**Yes**	No	21, 44, 69, 70
FPGS	rs4451422	No	NA	21
GGH	rs11545076	No	-	68
GGH	rs11545077	No	Yes	68
GGH	rs11545078	No	Yes	68
GGH	rs1800909	No	Yes	68
GGH	rs3758149	**Yes**	Yes	20, 44, 68
MTHFD1	rs1076991	No	NA	21
MTHFD1	rs1950902	No	NA	21
MTHFD1	rs2236225	**Yes**	No	21, 35, 71
MTHFD1	rs745686	No	NA	21
MTHFR	rs1801131	No	Yes	23, 25, 26, 27, 28, 33, 34, 45, 46, 52, 57, 71, 72, 75, 76, 79, 80

MTHFR	rs1801133	**Yes**	Yes | No*	23, 25, 26, 27, 28, 32, 33, 34, 35, 39, 42*, 43, 44, 45, 46, 47, 52, 57, 71, 72, 73, 74, 75, 76, 77, 78, 79, 80
MTR	rs10925257	No	NA	21
MTR	rs12759827	No	NA	21
MTR	rs1805087	No	-	21, 35, 57
MTR	rs2853523	No	NA	21
MTR	rs3768142	No	NA	21
MTR	rs4659724	No	NA	21
MTRR	rs10380	No	NA	21
MTRR	rs1532268	No	NA	21
MTRR	rs162036	No	NA	21
MTRR	rs1801394	**Yes**	No	21, 28, 35, 57
MTRR	rs2966952	No	NA	21
MTRR	rs326120	No	NA	21
MTRR	rs3776455	No	NA	21
SLC19A1	rs1051266	**Yes**	Yes* | No	20, 21, 22, 26*, 27, 28, 29*, 33, 34, 35, 38, 39, 40*, 41*, 42*, 43*
SLC19A1	rs1051296	**Yes**	Yes	67
SLC19A1	rs1131596	No	Yes	29, 38
SLC19A1	rs2838958	No	No	38
SLC19A1	rs3788200	No	Yes	29, 38
SLC19A1	rs4819128	No	NA	21
SLC19A1	rs61510559	**Yes**	No	23, 25, 45, 46, 47
SLC19A1	rs7499	No	NA	21
SLC22A6	rs10897310	No	No	29
SLC22A6	rs11231294	No	No	29
SLC22A6	rs3017670	No	NA	29
SLC22A6	rs4149172	No	No	29
SLC22A6	rs6591722	No	NA	29
SLC22A8	rs10792367	No	NA	29
SLC22A8	rs10897315	No	NA	29
SLC22A8	rs2187383	No	NA	29
SLC22A8	rs2276299	No	NA	21, 29
SLC22A8	rs3809069	No	NA	21, 29
SLC22A8	rs3948869	No	NA	29
SLC22A8	rs4149182	No	NA	29
SLC22A8	rs4149183	No	NA	21
SLC22A8	rs4963228	No	NA	29

SLC22A8	rs4963326	No	NA	29
SLC22A8	rs948980	No	NA	29
SLCO1A2	rs10505872	No	NA	29
SLCO1A2	rs10743413	No	NA	29
SLCO1A2	rs10770804	No	NA	29
SLCO1A2	rs10770805	No	NA	29
SLCO1A2	rs10841795	No	NA	29
SLCO1A2	rs10841803	No	NA	29
SLCO1A2	rs11045918	No	NA	29
SLCO1A2	rs11045919	No	NA	29
SLCO1A2	rs11045953	No	NA	29
SLCO1A2	rs11045961	No	NA	29
SLCO1A2	rs11045994	No	NA	29
SLCO1A2	rs11831407	No	NA	29
SLCO1A2	rs11837182	No	NA	29
SLCO1A2	rs12300594	No	NA	29
SLCO1A2	rs12319824	No	NA	29
SLCO1A2	rs16923597	No	NA	29
SLCO1A2	rs16923647	No	NA	29
SLCO1A2	rs2045938	No	NA	29
SLCO1A2	rs2045939	No	NA	29
SLCO1A2	rs2045940	No	NA	29
SLCO1A2	rs2306231	No	NA	29
SLCO1A2	rs2417977	No	NA	29
SLCO1A2	rs4148984	No	NA	29
SLCO1A2	rs4148988	No	NA	29
SLCO1A2	rs4149008	No	NA	29
SLCO1A2	rs4149009	**Yes**	No	27, 29, 48
SLCO1A2	rs4337089	No	NA	29
SLCO1A2	rs4762699	No	NA	29
SLCO1A2	rs4762818	No	NA	29
SLCO1A2	rs6487215	No	NA	29
SLCO1A2	rs7137767	No	NA	29
SLCO1A2	rs7301895	No	NA	29
SLCO1A2	rs7954757	No	NA	29
SLCO1A2	rs7962263	No	NA	29
SLCO1A2	rs7964783	No	NA	29
SLCO1B1	rs10444413	No	NA	22
SLCO1B1	rs10841753	**Yes**	Yes* | No	38, 49*, 50
SLCO1B1	rs10841769	No	NA	21
SLCO1B1	rs11045787	**Yes**	Yes	49

SLCO1B1	rs11045813	**Yes**	Yes	50
SLCO1B1	rs11045818	**Yes**	Yes* ǀ No	21, 49*, 50*
SLCO1B1	rs11045819	**Yes**	No	51, 21
SLCO1B1	rs11045821	**Yes**	No	50
SLCO1B1	rs11045823	No	NA	21
SLCO1B1	rs11045825	**Yes**	No	50
SLCO1B1	rs11045870	**Yes**	No	50
SLCO1B1	rs11045872	**Yes**	No	22, 49, 50
SLCO1B1	rs11045879	**Yes**	Yes* ǀ No	22, 25*, 29, 33, 35*, 49, 50*, 52
SLCO1B1	rs11045892	**Yes**	No	50
SLCO1B1	rs11045897	**Yes**	Yes	38, 53
SLCO1B1	rs1463565	No	NA	22
SLCO1B1	rs16923647	**Yes**	No	50
SLCO1B1	rs17328763	**Yes**	Yes	49
SLCO1B1	rs2169969	**Yes**	No	50
SLCO1B1	rs2306283	**Yes**	Yes* ǀ No	27*, 33, 38, 54
SLCO1B1	rs2417955	No	NA	22
SLCO1B1	rs2900476	**Yes**	No	22, 49
SLCO1B1	rs34671512	**Yes**	No	51
SLCO1B1	rs4149026	No	NA	22
SLCO1B1	rs4149034	No	NA	22
SLCO1B1	rs4149035	No	No	22, 29
SLCO1B1	rs4149056	**Yes**	Yes	21, 22, 27, 28, 32, 33, 34, 35, 38, 43, 49, 50, 51, 52, 54, 55
SLCO1B1	rs4149057	No	NA	22
SLCO1B1	rs4149076	**Yes**	No	49
SLCO1B1	rs4149081	**Yes**	Yes* ǀ No	22*, 25*, 49, 50*, 53*
SLCO1B1	rs4363657	No	NA	21
SLCO1B1	rs59502379	**Yes**	Yes	51
SLCO1B1	rs7966613	No	Yes	49
SLCO1B3	rs1002441	No	NA	29
SLCO1B3	rs1017385	No	NA	29
SLCO1B3	rs10841648	No	NA	29
SLCO1B3	rs10841660	No	NA	29
SLCO1B3	rs10841661	No	NA	29
SLCO1B3	rs10841697	No	NA	29
SLCO1B3	rs11045512	No	NA	29
SLCO1B3	rs11045573	No	NA	29
SLCO1B3	rs11045585	No	NA	29
SLCO1B3	rs11045598	No	NA	29

SLCO1B3	rs12824715	No	NA	29
SLCO1B3	rs1304608	No	NA	29
SLCO1B3	rs1549968	No	NA	29
SLCO1B3	rs1581194	No	NA	29
SLCO1B3	rs1966648	No	NA	29
SLCO1B3	rs2117032	No	NA	29
SLCO1B3	rs2417886	No	NA	29
SLCO1B3	rs2417940	No	NA	29
SLCO1B3	rs4149117	No	NA	29
SLCO1B3	rs4149121	No	NA	29
SLCO1B3	rs4149132	No	NA	29
SLCO1B3	rs4382961	No	NA	29
SLCO1B3	rs4762803	No	NA	29
SLCO1B3	rs7311358	No	NA	29
SLCO1B3	rs7962265	No	NA	29
SLCO1B3	rs7973653	No	NA	29
SLCO1B3	rs975657	No	NA	29
TYMS	rs1004474	No	NA	21
TYMS	rs151264360	No	NA	75
TYMS	rs2612100	No	NA	21
TYMS	rs2790	**Yes**	No	48
TYMS	rs2853533	No	NA	21
TYMS	rs2853741	No	NA	21
TYMS	rs34489327	**Yes**	No	25, 34, 43
TYMS	rs34743033	**Yes**	No	25, 33, 34, 42, 43, 46, 57, 71
TYMS	rs9967368	No	NA	21

* Indicates the studies that showed increased MTX exposure when there were conflicting reports for a variant

NA; Not applicable. The study genotyped for the variant, but did not present any pharmacokinetic data.

- Pharmacokinetic data was presented. No observable nor statistical differences between the genotypes
